# Pragmatic solutions to enhance self-management skills in solid organ transplant patients: systematic review and thematic analysis

**DOI:** 10.1186/s12875-022-01766-z

**Published:** 2022-06-30

**Authors:** Hamidreza Abtahi, Reza Safdari, Marsa Gholamzadeh

**Affiliations:** 1 Pulmonary and Critical care Medicine Department, Thoracic Research Center, Imam Khomeini Hospital Complex, Tehran University of Medical Sciences, Tehran, Iran; 2 Department of Health Information Management, School of Allied Medical Sciences, Tehran University of Medical Sciences, Tehran, Iran; 3 Ph.D. Candidate in Medical Informatics, Health Information Management Department, School of Allied Medical Sciences, Tehran University of Medical Sciences, Fardanesh Alley, 5th FloorQods Ave, Tehran, Iran

**Keywords:** Organ transplantation, Self-management, Solid organ, Patient-centered care, PRISMA

## Abstract

**Background:**

In organ transplantation, all patients must follow a complex treatment regimen for the rest of their lives. Hence, patients play an active role in the continuity of the care process in the form of self-management tasks. Thus, the main objective of our study was to investigate the pragmatic solutions applied by different studies to enhance adherence to self-management behaviors.

**Method:**

A systematic review was conducted in five databases from 2010 to August 2021 using keywords. Eligible studies were all English papers that developed self-management programs to enhance patient care in solid organ transplantation. The interventions were analyzed using thematic analysis to determine the main descriptive areas. The quality of the included articles was evaluated using the research critical appraisal program (CASP) tool.

**Results:**

Of the 691 retrieved articles, 40 met our inclusion criteria. Of these, 32 studies were devoted to the post-transplantation phase. Five main areas were determined (e-health programs for telemonitoring, non-electronic educational programs, non-electronic home-based symptom-monitoring programs, electronic educational plans for self-monitoring, and Telerehabilitation) according to thematic analysis. Most studies (72.5%) declared that developed programs and applied solutions had a statistically significant positive impact on self-management behavior enhancement in transplant patients.

**Conclusion:**

The results showed that an effective solution for improving organ transplantation needs patient collaboration to address psychological, social, and clinical aspects of patient care. Such programs can be applied during candidate selection, waiting list, and after transplantation by putting the patient at the center of care.

**Supplementary Information:**

The online version contains supplementary material available at 10.1186/s12875-022-01766-z.

## Background

There are several types of advanced illnesses that can lead to organ failure or organ dysfunction. Hence, solid organ transplantation has been considered the last therapeutic solution for end-stage diseases to improve survival [1]. Solid-organ transplantation (SOT) is not limited to a surgery in which a healthy organ is given to a person whose organ is disabled or not functioning properly. Indeed, it is a lifetime treatment option [2]. In organ transplantation, all patients must follow a complex treatment regimen, adherence to medication, a healthy lifestyle, and a special diet for the rest of their lives to prevent complications [3]. Thus, patient adherence to individual care plans is an efficient part of the transplantation process. Previous studies have also pointed to the active role of patients in the continuity of the care process in the form of self-management tasks [4].

Organ transplant patients are usually chronically ill patients who need long-term follow-up and daily self-management care [5–7]. Self-management refers to a patient’s ability to manage his/her daily symptoms properly and cope with a lifestyle change, and physical and psychological status in collaboration with his family and healthcare professionals [8]. The patient should be placed at the center of the organ transplantation care process to enhance self-management tasks [9, 10]. Thus, self-management programs can be implemented using patient-centered care approaches for organ transplantations.

Evidence has shown that self-management behaviors improve post-transplant survival, medication adherence, quality of life, and physical activities [11]. Some solutions to improve self-management behavior are crucial for better outcomes [12]. In this regard, Bittermann [13] believed high-quality evidence-based medical care without involving a patient in his/her care would not guarantee transplant success. Thus, various approaches have been employed to enhance self-management in solid organ transplantation. Despite the existence of various models for self-management care for chronic care [14, 15], no conceptual framework or systematic review has been devised in terms of solid organ transplantation.

The main objective of our study was to investigate the pragmatic solutions in solid organ transplantation to enhance patient collaboration to address psychological, social, and clinical aspects of patient care in form of self-management programs. Specific aims of this survey are as follows (1) recognizing the main themes and sub-themes of various pragmatic solutions regarding self-management in organ transplantation patients; (2) representing an overview of employed solutions and their characteristics; (3) summarizing common features of self-management programs; and (4) specifying the outcomes of such programs.

### Main concept and related terms

Since self-management as the main idea of our study is a broad concept, we describe this term from our point of view in this section. According to Matarese et al. [16], substitute terms for self-management are self-care, self-care management, disease management, management of treatment regimens, and illness management. According to the World Health Organization (WHO), self-care refers to any activity a patient does to stay healthy [17]. While Self-management defines as the ability of a person to properly cope with their physical and mental condition and lifestyle changes along with a chronic illness in collaboration with healthcare providers”, self-care can happen either in the presence or absence of healthcare professionals [18]. A recent study by Kongsted et al. (2021) reports that self-care is a broad term that can cover the self-management concept [19]. Self-management of the chronic disease comprises symptom management, medication adherence, and being healthy is part of self-care action and should be conducted in collaboration with healthcare providers. Hence, we focus on Kongsted’s definition in our article.

Regarding solutions and interventions were employed to improve the health status of patients, these kinds of interventions could take place in form of some tasks and skills that needed to be done by patients to improve their health status in collaboration with health care providers which are named self-management techniques, tasks, or behaviors in literature. Investigating pragmatic solutions to enhance self-management in organ transplantation systems was the main focus of this research. Self-management task or behavior are other terms utilized in this context. They refer to the daily actions or practices that must be performed by the patient to be in the best possible physical and mental condition, in addition to adhering to the treatment.

## Methods

A systematic search of four databases (Web of Science, Scopus, PsycInfo through Medline OVID, and Medline PubMed, Cochrane Library, IEEE (Institute of Electrical and Electronics Engineers), ScienceDirect) was conducted from 2010 to August 2021 using keywords alongside Mesh terms (Additional file, Table A-1). Also, the original articles before 2010 with more than five citations have been retrieved. These databases were selected for inclusion in qualitative studies and health research. The keywords used in the search strategy were drawn from preliminary searches according to the goals of our study. These keywords were validated, and additional keywords were added by checking the terms used in the articles identified in the preliminary searches. Boolean search strategies are described in Table A-1 in the Additional file in Table A-1. Since no result was found in the IEEE and Cochrane databases, they were removed from the source databases. Articles were retrieved from the databases according to our search strategy. Next, related articles were added using a simple search in Google Scholar and reference checking manually. We utilized EndNote software for resource management. This systematic review was completed according to the Preferred Reporting Items for Systematic Reviews and Meta-Analyses (PRISMA) checklist to ensure the inclusion of relevant studies [20].

### Inclusion and exclusion criteria for study selection

The research questions and inclusion criteria were developed based on PCC (Population or participants, Concept, Context) for qualitative review studies [21]. The population referred to any patients in the transplant system who were advantaged from organ transplantation. Our population includes patients in all phases of organ transplantation who are candidates for organ transplantation, those on the waiting list, and organ recipients. The concept is referred to the self-management. Context referred to any action, solution, or intervention that can help and engage patients to improve their health and cope with the disease.

Matarese et al. believed that a self-management term is used in the medical domain while self-care is used in nursing literature [16]. According to Matarese’s suggestion, all related keywords to the self-management concept were considered to find all studies conducted to improve self-care behaviors.

Articles were included if they met the following criteria: 1) The focus of the study was on applying self-management solutions through the transplantation processes, 2) Patients in any phase of solid organ transplantation; 3) This study covered all phases of solid organ transplantation, 4) Published in the past 11 years, 5) Patients aged > 18 years, 6) Peer-reviewed, 7) Limited to those published in the English language, 8) Only published articles and reviews in peer-reviewed journals were included, 9) All types of study and designs, including descriptive studies, feasibility, or development solutions, 10) Solid organ transplantation including heart transplantation, heart–lung transplantation, lung transplantation, kidney transplantation, liver transplantation.

Articles excluded if they met the following criteria: 1) Unrelated title, abstract, or full text of the article to the application of self-management in organ transplantation, 2) Thesis, book chapters, letters to editors, short briefs, reports, technical reports, book reviews, reviews, or meta-analyses were not considered; 3) non-English papers; 4) Studies on blood donation, stem cell transplantation, tissue transplantation or studies related to animal studies were excluded.

### Study screening selection phase

**Fig. 1:**
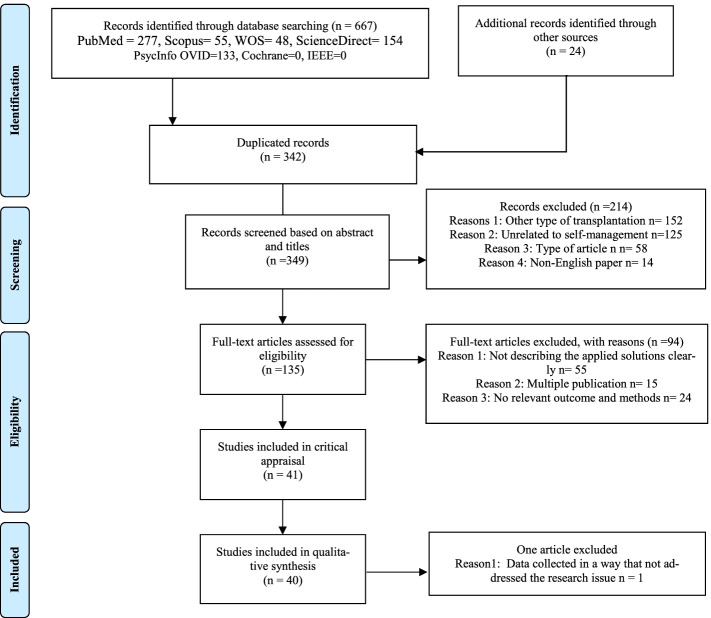
The flow diagram of PRISMA

The design of our study followed the 27-item checklist of Preferred Reporting Items for Systematic Reviews and Meta-Analyses’ (PRISMA) statement [22]. Thus, the PRISMA flow diagram to screen articles is represented in Fig. 1.

After that, duplicate articles were removed. The first stage included a screening of titles and abstracts based on research questions and inclusion criteria by the first author. At the same time, a second reviewer screened studies randomly. Next, the full texts of relevant studies were investigated by two reviewers thoroughly based on our inclusion criteria. All of the papers that met our inclusion criteria were considered for qualitative analysis. Data extraction forms were designed to facilitate the analysis of reviewed studies. The extraction form was filled based on predefined classifications to diminish bias by two authors independently. The next reviewer assessed and verified the extracted data.

### Critical quality appraisal

The methodological quality of the included articles was evaluated using the qualitative research critical appraisal program (CASP) tool by two authors. This instrument is frequently used in systematic reviews for qualitative synthesis [23]. It was employed to appraise the strengths and limitations of any qualitative research methodology. It is recommended for health-related research and is appropriate for novice researchers [24]. Critical appraisal was performed by two researchers independently.

### Analysis

Specific categories were considered to classify and analyze relevant articles. All articles were synthesized concerning general and specific domains based on categories. Descriptive statistical analysis and framework suggestions were conducted based on these predefined categories.

Due to the heterogeneity of papers, conducting a meta-analysis is impossible. Thus, a thematic analysis was conducted to find the main concepts regarding self-management solutions undertaken in the organ as a qualitative analytic method. Thematic analysis helps to discover the main concept in the articles regarding the research question by finding frequent keywords in included articles [25]. In addition, a thematic analysis could find the best classification for the applied solutions. Thus, all extracted data were coded and classified to extract the main themes and key elements.

Descriptive themes were identified based on Tomas and Harden's technique [26]. First, full texts of eligible articles were imported to ATLAS.ti® Software. Then, line-by-line coding of all articles was conducted by two reviewers independently [27]. Codes were derived from the hidden concepts in articles through the deductive process. The extracted codes were validated by a third reviewer. Next, all of the similar codes were merged and grouped. Then, one reviewer (MG) linked extracted codes to identify underlying themes. Other authors validated the main themes and sub-themes. Last, a thematic map was devised under expert consultation in an iterative process.

Since content analysis did not represent the effectiveness of developed programs, another approach was employed. The effectiveness of applied solutions was investigated by reviewing the outcomes of studies. The outcome measures were classified into three categories, clinical, patient aspect, and user perspectives. The clinical outcome domain is used to quantify or describe the clinical effect of the transplantation, such as readmission to the hospital. We utilized the Sign test to assess the effect of proposed solutions in either direction (e.g., positive or negative) for clinical, patient aspect, and user perspective outcomes. The effectiveness assigned to each study was determined according to a significant level of outcome measures. The effect of interventions was defined as (1) plus positive or effective (i.e., statistically significant *P*_Value_ < 0.001) (2) positive or to some extent (i.e. statistically significant *P*_Value_ < 0.05), and (3) no effect or negative (i.e. not statistically significant). If they did not declare the significant level, the effectiveness assigned to not clear.

## Results

A systematic search in electronic databases yielded 691 citations, of which 342 studies were duplicated. Subsequently, 325 papers were screened based on their titles and abstracts. Later, 105 articles were excluded because of their irrelevance in abstract screening. Next, the full text of 220 articles was screened. Ultimately, 45 articles remained. After a quality appraisal, 40 studies were eligible. The screening process for articles based on the PRISMA checklist is shown in Fig. 1.

All included studies had a minimum score (10 out of 20) of quality assessment using the CASP tool. Only five papers were excluded based on the quality appraisal assessment. Therefore, forty articles were identified as eligible for the qualitative analysis.

### General characteristics of articles

Next, 40 retrieved studies were analyzed thematically. The extracted data from these papers are summarized in detail in tabular form for further analysis. The analysis indicated that developing self-care programs for transplant patients showed an upward trend in the last ten years. Most eligible studies were recently published (11 articles in 2020). There were 16 RCTs, 15 descriptive studies, four cross-sectional studies, three cohort studies, and two before-after studies. The average sample size of participants was 87 (8–540), and the median follow-up duration was six months (2 –36 months). A large portion of the articles (51.22%) originated in the Americas, while only 26.83% of them belonged to the European continent. Finally, 19% of the articles were published in Asian countries. Concerning the transplantation phase, the majority of studies devoted to the post-transplantation phase with 33 studies (79.48%) to improving self-management behavior (Table 1).

**Table 1:** General characteristics of studies

Year	Frequency
2020–2021	15
2017–2019	14
2014–2016	6
2010–2013	5
2020–2021	15
**Country of origin**	**Frequency**
USA	16
Canada	5
Germany	3
China	2
Netherlands	2
Norway	2
Taiwan	2
South Korea	2
Australia	1
Belgium	1
Denmark	1
Iran	1
Spain	1
UK	1
**The phase of transplantation and transplantation type**	**Frequency**
**For transplant candidates and donors**	**1**
Kidney	1
**Posttransplant patients**	**32**
Any solid organ	3
Heart	3
All organ recipients except lung recipients	1
Kidney	12
Kidney and Liver	1
Liver	4
Lung	7
**Both post-transplant patients and candidates**	**4**
Any solid organ	1
Kidney	2
Lung	1
**For transplant candidates**	**3**
Kidney	1
Liver	2

### Applied solutions and approaches to enhance self-management tasks

**Fig. 2:**
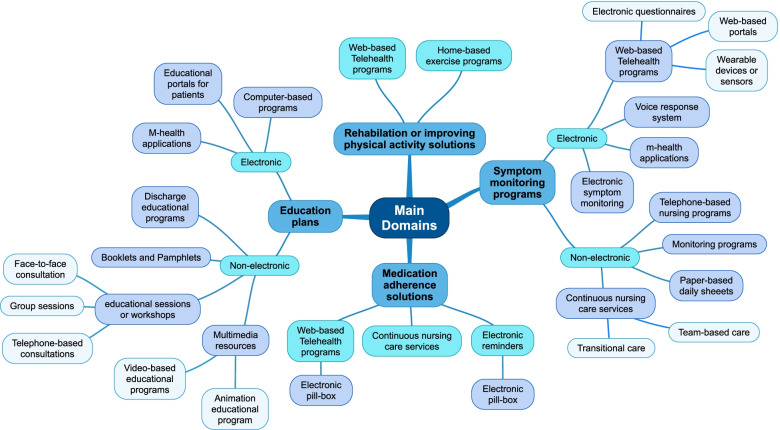
The identified main theme and sub-theme from literature

Since the designs of studies were diverse, a “thematic analysis” as a common qualitative content analysis technique was employed to extract the foremost themes and concepts. Accordingly, applied solutions were classified into four main themes and 42 sub-themes based on the content analysis. The tree-based structure of themes is shown in Fig. 2. We categorized all the approaches in the reviewed articles into five main categories based on findings. Each category is described in the following.

- **e-Health programs for telemonitoring with 19 studies (47.5%):** In two articles, patients received medical wearable devices for self-monitoring at home [28, 29]. Patients are asked to measure their signs and symptoms as usual during home monitoring in this category. These types of interventions are called remote patient symptom monitoring programs. In nine studies, the authors employed a mobile-based application to monitor patient signs and symptoms, enhance medication adherence, and send reminders or alerts [30–38]. In one study, a home-based remotely monitored intervention using wearable accelerometer devices was employed to promote post-transplant physical activity in patients [39]. In another study, an interactive voice response system was developed to enhance self-management behaviors in kidney transplant recipients [40]. In six studies, researchers developed web-based portals to enhance patient care and symptom monitoring via electronic questionnaires and forms [41–46]. In this category, programs exchange patient information with healthcare providers periodically or in real-time.
- **Non-electronic educational programs with eight studies (20%):** Educating organ transplant patients to cope with their situation is critical in transplantation programs [47]. Multimedia-based programs to educate transplant patients were employed in four papers. Two studies created educational animation programs to improve self-management behaviors in kidney transplant patients in two different formats [48, 49]. Video-based programs were developed in two studies to improve medication adherence and symptom management in renal transplantation [50, 51]. In the other three studies, face-to-face educational sessions, telephone-based consultations, and educational booklets were used to educate transplant patients and improve patient knowledge regarding organ transplantation [52–54]. A structured teaching program at discharge time is another solution to improve self-management tasks among organ transplant patients [55].
- **Non-electronic home-based symptom-monitoring programs with six studies (15%):** In one study, a self-management tool in the form of a paper-based diary sheet was developed for daily self-observation [56]. In three studies, nurse-led self-management programs were implemented to enhance organ transplant patient care. The nurses monitor, educate, and consult patients using telephone or email through continuous nursing service care [57–59]. In two other studies, transplant care team members used team-based interventions to empower self-care patient behaviors. In one study, support groups were used to educate and monitor patients at regular meetings [60]. A cross-age peer mentoring program was applied to support transplant patients in monitoring symptoms. The result of this intervention was associated with meaningful improvement in self-management adherence behaviors [61].
- **Electronic educational plan or self-monitoring with five studies (12.5%):** In one article, researchers developed web-based portals to provide patients with customized educational content using the patient’s electronic file for each lung transplant recipient [62]. In two studies, they developed a computer-based educational program for window applications to educate patients regarding organ transplantation [63, 64]. In another study, a mobile medication manager application was developed to educate patients regarding medication adherence [65]. Wickerson et al. developed a web-based portal to educate patients by a virtual nurse. [66]. In this category, the developed programs did not exchange any information with healthcare providers or transplant care teams.
- **Tele-rehabilitation with two studies (5%):** Two studies developed Telerehabilitation programs for lung and liver transplant candidates as home-based exercise programs. Wickerson et al. developed a web application during the COVID-19 pandemic to adjust oxygen prescription and monitor home-based rehabilitation in lung transplant recipients and candidates [67]. A daily home-based exercise program (HELP) was developed in another study to overcome frailty problems in liver transplantation candidates [68].

Accordingly, these interventions were implemented on different platforms. The analysis showed that smartphones and tablets have a high frequency among other platforms, while paper-based sheets have the lowest number. All of the applied platforms and a summary of reviewed studies are shown in Table 2.

**Table 2:** The summary and characteristics of interventions applied in reviewed articles

#	Author	Year	Study	Type of intervention	Type of program	Technology platform	Organ Tx	Stage of transplantation	Country	Effectiveness	CASP Score (From 20)
1	Schenkel. F et al. [28]	2019	Cohort	ehealth programs for telemonitoring	Electronic symptom monitoring	Bluetooth enabled device, Tablet,	Lung	Posttransplant	USA	Not clear	18
2	Serper, M. et al.[39]	2020	RCT	ehealth programs for telemonitoring	Telemonitoring with telephone and email	Telephone, email, wearable accelerometer devices	Kidney and Liver	Posttransplant	USA	To some extent	16
3	Lieb.M. et al.[43]	2020	single-center prospective observational trial	ehealth programs for telemonitoring	Web-based telehealth program	multicompartment pillbox, web portals	Heart	Posttransplant	Germany	Not clear	18
4	Schaevers, V. et al.[62]	2012	Descriptive	Electronic education plan	An educational portal for patients	Web Portals	Lung	Posttransplant	Belgium	Effective	19
5	Wickerson, L. et al.[67]	2021	Descriptive	Telerehabilitation	Web-based telehealth program	Pulse Oximeter, remote monitoring app, exercise equipment	Lung	Posttransplant and candidates	Canada	Effective	18
6	Evald, L. et al.[56]	2020	Descriptive	Non-electronic Home-based monitoring program	Paper-based diary program	A printed diary sheet	Lung	Posttransplant	Norway	Effective	19
7	Chen, Y. W. et al.[30]	2020	Descriptive	ehealth programs for telemonitoring	mhealth application	Smartphones	Heart	Posttransplant	Taiwan	Not clear	17
8	Kim, S. et al.[50]	2020	Descriptive	Non-electronic educational program	Multimedia education	Smartphones or tablet	kidney	Posttransplant	South Korea	Effective	16
9	Li, L. et al.[59]	2020	Descriptive	Non-electronic Home-based monitoring program	Continuous nursing service care	Telephone	kidney	Posttransplant	China	Effective	16
10	Kayler, L. K. et al.[49]	2020	Descriptive	Non-electronic educational program	Multimedia education	Smartphones or tablet	kidney	Candidates and donors	USA	Effective	17
11	Kayler, L. K. et al.[48]	2020	pre-post study	Non-electronic educational program	Multimedia education	Smartphones or tablet	kidney	Transplant candidates	USA	Effective	18
12	Lerret.S et al.[35]	2021	RCT	ehealth programs for telemonitoring	mhealth application	Smartphones or tablet	All organ recipients except lung	Posttransplant	USA	Effective	19
13	Nielsen, C. et al.[44]	2020	Descriptive	ehealth programs for telemonitoring	Web-based telehealth program	Smartphones or tablet, Portals	Kidney	Posttransplant	Denmark	Not clear	16
14	Leek, R. B. et al.[53]	2019	prospective cohort	Non-electronic educational program	education sessions, pamphlets, or booklets	Face to Face Sessions or telephone-based consultation	Liver	Posttransplant	USA	Effective	20
15	Hickman, I. J. et al.[52]	2019	Qualitative study	Non-electronic educational program	education sessions, pamphlets, or booklets	Face to Face Sessions or telephone-based consultation	Liver	Posttransplant	Australia	Effective	16
16	Mansell, H. et al.[51]	2019	RCT	Non-electronic educational program	Multimedia education	Smartphones or tablet	Kidney	Posttransplant	USA	Not clear	14
17	Van Lint, C. et al.[29]	2017	RCT	ehealth programs for telemonitoring	Electronic symptom monitoring	Smartphones or tablet, Portals	Kidney	Posttransplant	Netherlands	Effective	15
18	Wang, W. et al.[46]	2017	Qualitative study	ehealth programs for telemonitoring	Web-based telehealth program	Smartphones or tablet, Portals	Kidney	Posttransplant	Netherlands	Effective	20
19	Bailey, D. et al.[57]	2017	RCT	Non-electronic Home-based monitoring program	Continuous nursing service care	Telephone	Liver	Transplant candidates	USA	Not effective	18
20	Rosenberger, E. M. et al.[37]	2017	RCT	ehealth programs for telemonitoring	mhealth application	Smartphones or tablet, Portals	Lung	Posttransplant	USA	Effective	20
21	Jiang,Y. et al.[34]	2016	Cross-sectional	ehealth programs for telemonitoring	mhealth application	Smartphones or tablet, Portals	Lung	Posttransplant	USA	Effective	16
22	DeVito Dabbs, A. et al.[31]	2016	RCT	ehealth programs for telemonitoring	mhealth application	Smartphones or tablet, Portals	Lung	Posttransplant	USA	Effective	18
23	Hsiao, C. Y. et al.[60]	2016	RCT	Non-electronic Home-based monitoring program	Team-based care program	Face to Face Sessions or telephone-based consultation	Kidney	Posttransplant	Taiwan	Effective	18
24	McGillicuddy, J. W. et al.[36]	2015	RCT	ehealth programs for telemonitoring	mhealth application	Smartphones or tablet	kidney	Posttransplant	USA	Effective	15
25	Jerson, B. et al.[61]	2013	Qualitative study	Non-electronic Home-based monitoring program	Team-based care Mentoring program	Face to Face Sessions or telephone-based consultation	Liver	Posttransplant	USA	Effective	15
26	Urstad, K. H. et al.[54]	2012	Randomized controlled	Non-electronic educational program	education sessions, pamphlets, or booklets	Face to Face Sessions or telephone-based consultation	Kidney	Posttransplant	Norway	Effective	17
27	Lieb, M. et al.[42]	2020	observational study	ehealth programs for telemonitoring	Web-based telehealth program	Electronic pillbox, web portals	Kidney	Posttransplant and candidates	Germany	Effective	17
28	Been-Dahmen, J. et al.[58]	2019	Cross-sectional	Non-electronic Home-based monitoring program	Continuous nursing service care	Telephone	Kidney	Posttransplant and candidates	Canada	Effective	15
29	Harrison, J. J. et al.[64]	2017	RCT	Electronic education plan	Computer-based educational program	Smartphones or tablet	any organ	Posttransplant	Canada	Not clear	15
30	Korus, M. et al.[41]	2015	Descriptive	ehealth programs for telemonitoring	Web-based telehealth program	web portals	any organ	Posttransplant	Canada	Effective	15
31	Frank-Bader, M. et al.[55]	2011	Descriptive	Non-electronic educational program	Training educational program at discharge	Face to Face Sessions or telephone-based consultation	any organ	Posttransplant and candidates	USA	Effective	17
32	Shellmer, D et al.[38]	2016	Descriptive	ehealth programs for telemonitoring	mhealth application	Smartphones or tablet	any organ	Posttransplant	USA	Effective	18
33	Ganjali, R et al.[40]	2019	RCT	ehealth programs for telemonitoring	Voice response system	Telephone	Kidney	Posttransplant	Iran	Not clear	18
34	Williams, F et al.[68]	2019	Observational study	Telerehabilitation	Home-based exercise program	Telephone	Liver	Transplant candidates	UK	Effective	18
35	Geramita, E et al.[32]	2020	RCT	ehealth programs for telemonitoring	mhealth application	Smartphones or tablet	Lung	Posttransplant	USA	Not clear	17
36	Freier, C et al.[63]	2010	Before-after	Electronic education plan	Computer-based educational program	Smartphones or tablet	Kidney	Posttransplant	Germany	Effective	17
37	Han, Ahram, et al.[65]	2019	RCT	Electronic education plan	mhealth application	Smartphones or tablet	Kidney	Posttransplant	Korea	Not effective	20
38	Tian M et al.[45]	2021	Cohort	ehealth programs for telemonitoring	Web-based telehealth program	Web Portals	Liver	Posttransplant	China	Effective	20
39	Gomis-Pastor, M et al.[33]	2021	Descriptive	ehealth programs for telemonitoring	mhealth application	Smartphones or tablet	Heart	Posttransplant	Spain	Effective	17
40	Côté, J. et al.[66]	2019	RCT	Electronic education plan	Interactive Web-based sessions hosted by a virtual nurse	Web-Portals	kidney	Posttransplant	Canada	Effective	17

### Most common features and modules of self-management interventions

**Fig. 3:**
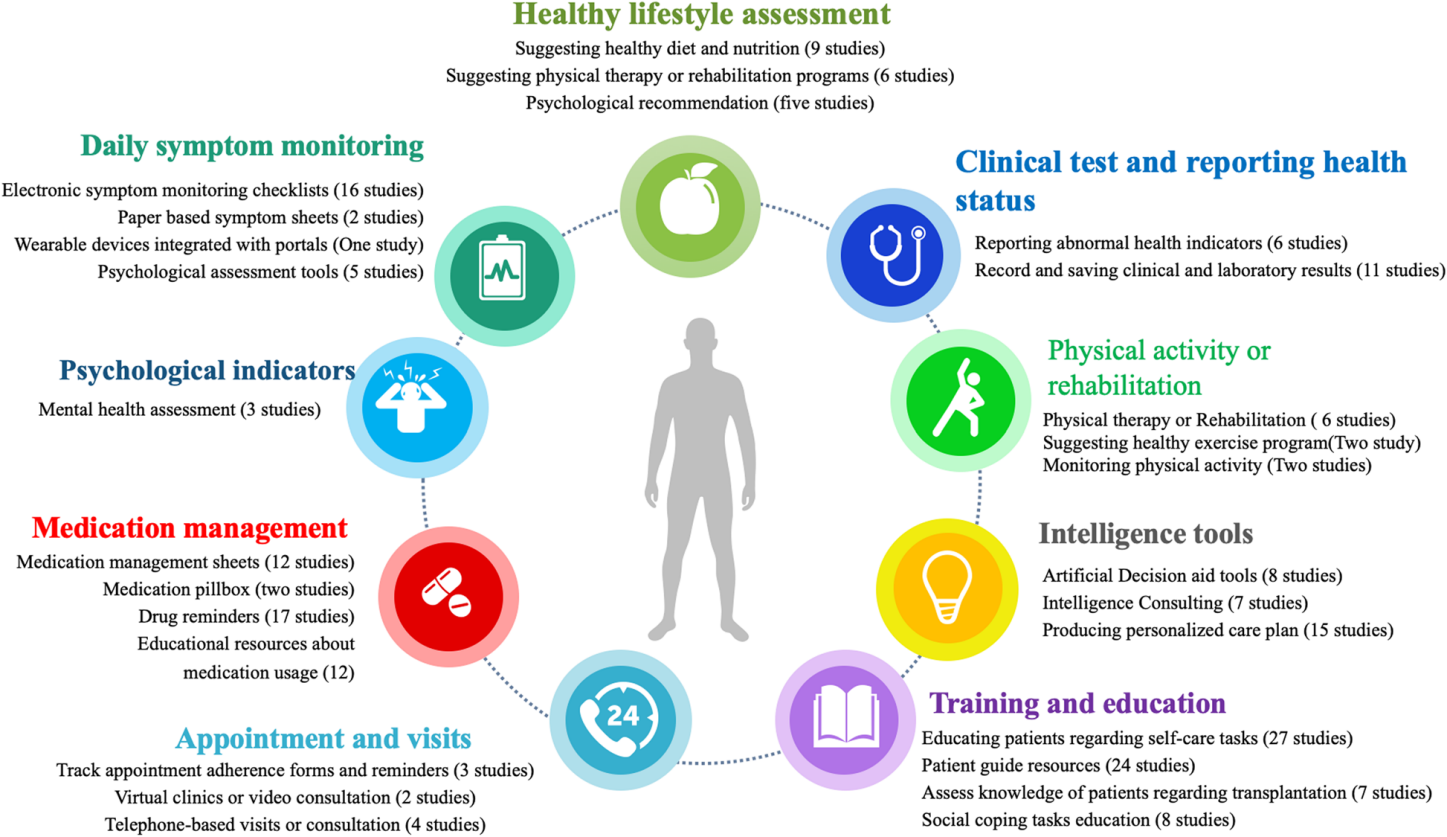
Most common features and modules of self-management interventions (Icon’s source: www.flaticon.com)

The developed programs utilized different solutions to enhance self-management behaviors and engage transplant patients to follow a series of self-management tasks. Such interventions have various characteristics. These characteristics comprise nine categories including daily symptom monitoring, medication management section, appointment, and visit modules, reporting and saving data, applying intelligence tools, suggesting a healthy lifestyle module, physical activity management and rehabilitation, psychological indicators, and training and educating features. Of these, symptom monitoring and educating patients are the most common among the identified features and capabilities. Different aspects of applied self-management interventions are summarized in the infographics in Fig. 3. Accordingly, the frequency of the features in each domain is described in Fig. 3.

Moreover, various questionnaires were applied to the reviewed articles to assess the patients in the form of checklists, electronic forms, or paper-based questionnaires. The analysis showed that the researchers preferred to use standard and valid questionnaires instead of self-administered questionnaires. Such checklists and questionnaires tried to assess different patients’ psychological, behavioral, or physical status, as well as the quality of life of patients. The questionnaires are presented in Table 3.

**Table 3:** Most used standard and valid questionnaires

#	Name	Description	Count
1	**Beliefs about medicines using the Beliefs about Medicines Questionnaire (BMQ)**	Two five-item scales to assess the patient’s belief regarding the need for prescribed medication for controlling their disease and their concerns about adverse side effects	**4**
2	**World Health Organization Quality of Life Scale (WHOQOL-BREF)**	It was developed by the World Health Organization (WHO), as a short form of WHOQOL-100. It covers all aspects of the QOL (quality of life) scales including physical health, psychological, social relationship, and environment	**4**
3	**Basel Assessment of Adherence to Immunosuppressive Medication Scale (BAASIS©)**	It is used as a medication adherence measurement scale in transplant recipients	**3**
4	**Patient Health Questionnaire (PHQ-9)**	It is a kind of easy-to-use patient questionnaire as a self-administered version of the PRIME-MD diagnostic instrument for common mental disorders. It can measure the severity of depression	**2**
5	**Brief Perceived Social Support Questionnaire (F-SozU K-6)**	The 6-item five-point Likert scale brief version of F-SozU to measure general perceived social support. Higher scores display higher levels of perceived social support	**2**
6	**Relationships Scales Questionnaire (RSQ)**	It is used to evaluate the psychometric properties of the relationship scales questionnaire (RSQ)	**2**
7	**Medication Experience Scale for Immunosuppressants (MESI)**	It is a seven-item self-report questionnaire to evaluate subjective experiences and attitudes toward immunosuppressive medication among patients	**2**
8	**Transplant Effect Questionnaire (TxEQ-D)**	It was used to evaluate the specific problems associated with organ transplantation in five subscales such as “worry”, “guilt”, “disclosure”, “responsibility”, and “adherence”	**2**
9	**Satisfaction with Information about Medicines Scale (SIMS-D)**	The SIMS-D assesses patients’ satisfaction with information about safe and accurate self-management of medicines	**2**
10	**The Short Form 36 Health Survey Questionnaire (SF-36) or 12-item Short-Form Health Survey (SF-12)**	It is a self-reported measure of health and quality of life status for a specific disease population. It is available in multiple languages	**2**
11	**Mishel Uncertainty in Illness Scale (MUIS)**	It examined the impact of uncertainty on illness	**2**
12	**The Center for Epidemiological Studies-Depression (CES-D)**	It is a 20-item questionnaire to estimate how often they experienced symptoms associated with depression over the past week	**2**
13	**Hospital Anxiety and Depression Scale (HADS)**	It is a 14-items questionnaire to assess generalized anxiety disorder and symptoms of depression	**2**
14	**General Self-Efficacy (GSE) scale**	It is a 10-item self-report questionnaire to assess optimistic self-beliefs to cope with a variety of difficulties in life	**2**
15	**Exercise of Self-care Agency (ESCA) Scale**	It assesses one's ability for self-care in different areas comprising self-concept, self-responsibility, knowledge and information seeking, and passivity	**2**
16	**Self-Efficacy for Managing Chronic Disease 6-Item Scale (SES6C)**	It is a free scale to assess how confident patients have with chronic illness in performing certain activities on a visual analog scale	**2**
17	**Health Education Impact Questionnaire (HEIQ)**	It is a patient-specific questionnaire to assess the effectiveness of patient education programs	**2**
18	**EuroQol-visual analogue scales (EQ-VAS)**	It is used to describe the level of patients’ health problems in five dimensions	**2**
19	**Self-Efficacy for Exercise (SEE) Scale**	A 9-item questionnaire to assess the confidence level of participants	**1**
20	**Long-Term Medication Behavior Self-Efficacy Scale (LTMBSES)**	It is designed to assess the self-efficacy behavior of transplant recipients regarding long-term medication	**1**
21	**Treatment Adherence Measure (TAM)**	It is a seven-item questioning item to assess a patient's adherence to treatment	**1**

### Effectiveness of applied solutions

**Fig. 4:**
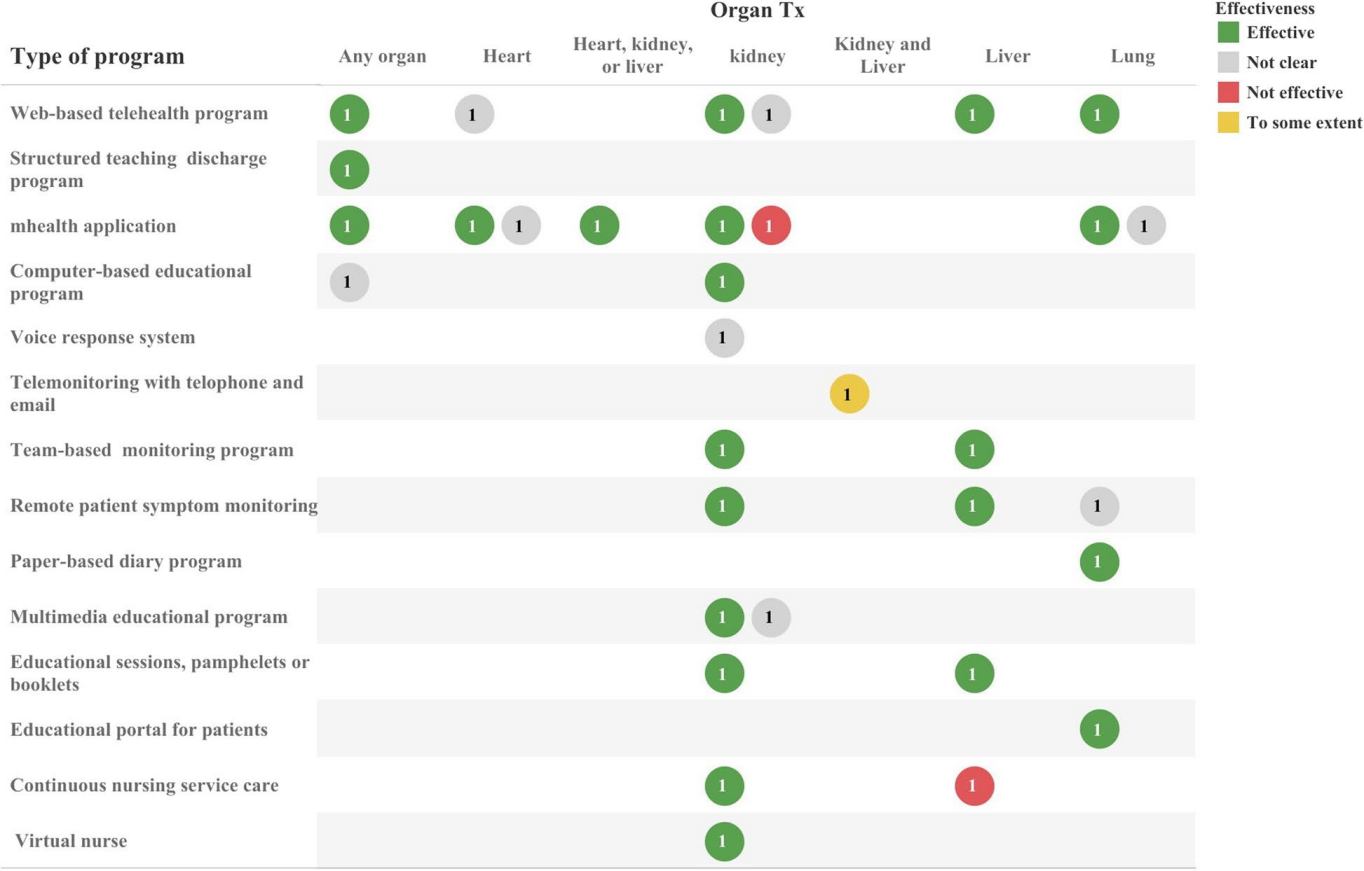
The effectiveness of developed programs

The effectiveness of the applied solutions and implemented programs were evaluated in the reviewed articles based on different outcome measures. Overall, the impact of designing different solutions to improve self-management tasks was significantly positive in 29 studies (72.50%), while two studies (5%) declared that applied solutions were not effective in improving self-management behaviors; in one study (2.50%), the intervention was effective to some extent. Accordingly, eight studies (20%) believed that the applied solutions may be useful, but the effectiveness of the developed programs was not clear. The effectiveness of the developed program in improving self-management tasks in transplant patients is shown in Fig. 4 in terms of program type and transplantation organ.

### Outcome measures

The impact of applied interventions on self-management tasks was evaluated using various outcome measures and metrics. These outcome measures can be divided into three main categories: clinical outcome measures related to transplantation outcomes, outcome measures related to self-management behavior of patients, and outcome measures related to system usage.

Outcome measures devoted to transplantation outcomes included readmission rate (eight studies) [31, 32, 34, 35, 45, 62, 65, 68], abnormal health indicator reports (12 studies) [28, 31, 34, 37, 40, 53, 57, 60, 62, 65, 68], survival rate (six studies) [31, 34, 37, 45, 62, 64], organ rejection (six studies) [32, 35, 37, 54, 64, 65], episodes of infection (two studies) [35, 60], unplanned returns to the operating room (two studies) [37, 68], and hospital charge (three studies) [28, 45, 51].

Metrics related to self-management tasks and behaviors of patients included self-efficacy (25 studies) [31–35, 37, 38, 40, 42, 46, 48, 49, 51, 54, 57, 58, 61, 63, 64, 66–69], medication adherence (19 studies)[29, 31, 33, 36, 38, 40–42, 51, 55, 58, 61, 62, 64–66], level of patient satisfaction (28 studies) [29, 31, 34, 35, 37, 38, 40, 42, 43, 49–51, 54, 56–58, 60–68], adherence to appointments and days in hospital (11 studies) [29, 30, 37, 42, 49, 51, 55, 56, 63, 65, 66], physical activity (15 studies) [28, 33, 34, 36, 39, 40, 42, 43, 54, 58, 61, 62, 67, 68, 70], patient's knowledge regarding self-management tasks (21 studies) [31, 33, 40, 41, 43, 46, 48, 49, 51, 53, 55, 57–61, 63, 64, 66, 69, 70], quality of life (24 studies) [28, 31, 33, 35, 38–40, 42, 43, 46, 51, 52, 54, 57, 58, 60–65, 68], clinical symptoms and indicators such as the results of clinical tests (6 MWT, GFR, Cr, SpO2,HR, FEV1 and etc.) (26 studies) [28, 29, 32–34, 37–39, 42–45, 52–54, 56–65, 67, 68], self-care behavior scale (15 studies) [31, 33–35, 37, 46, 50, 52, 56–58, 60, 64–66], emergency visits rate (three studies) [62, 67, 68], self-care agency level (18 studies) [29, 31, 32, 34, 37, 43, 46, 48, 52, 54, 56, 58–61, 64, 65, 69], empowerment scale (two studies) [46, 60], socio-demographic factors (10 studies) [31, 37, 54, 58, 61, 64, 65, 68], mental health indicators (11 study) [32–34, 37, 42, 54, 57, 58, 65, 68].

Metrics related to system and program usage included ease of use (22 studies)[28, 32–35, 38, 40, 41, 44, 46, 49, 53, 58, 59, 61, 63, 65–69], usefulness (24 studies) [28, 29, 33–35, 38, 40, 41, 44–46, 48, 53, 58, 59, 63, 65–67], usage rate (nine studies) [33, 38, 40, 41, 55, 58, 64–66], trustiness or reliability (11 studies) [28, 29, 32, 37, 41, 44–46, 48, 49, 61], adherence to system recommendation (10 studies) [28, 29, 33, 38, 41, 45, 65, 67, 68], acceptability (21 studies) [29, 33–35, 38, 40, 41, 45, 46, 48, 49, 53–55, 58, 61, 65, 66], and intention to use (17 studies) [29, 30, 34, 35, 37, 38, 40, 41, 48, 49, 58, 61, 64, 65, 67]. All of these indicators, based on their effectiveness in the reviewed studies, are described in Table 4.

**Table 4:** Effectiveness of outcome measures in reviewed studies

*Main domains*	*Outcome/Metrics*	*Effectiveness*			
		**Positive**	**To some extent**	**Not clear**	**Negative**
***Clinical outcomes in transplantation outcomes in per month***	Readmission	5		2	1
	Abnormal health indicators report	8		2	2
	Survival rate	3		2	1
	Acute organ rejection	3		2	1
	Episodes of getting an infection	2			
	Unplanned returns to operating room	2		2	
	Hospital charges	1		2	
	Clinical symptoms and indicators (6MWT, GFR, Cr, spo2, HR, etc.)	19	1	5	1
***Patient’s aspect***	Self-efficacy	18		5	2
	Medication adherence	13		5	1
	Level of patient satisfaction	21		5	2
	Adherence to appointments and days in the hospital	8		2	1
	Physical activity	11	1	3	
	Patient's knowledge regarding self-management tasks	16		4	1
	Quality of life	16	1	5	2
	Self-Care Behavior Scale	12		1	2
	Emergency visits	2		1	
	Self-care agency level	14		3	1
	Empowerment Scale	2			
	Mental health status	8		1	2
	Socio-demographic factors	8		1	1
***Outcomes related to users***	Ease of Use	17		4	1
	Usefulness	18		5	1
	Usage rate	6		2	1
	Trustiness	8		3	
	Intention to Use	13		3	1
	Acceptability	18		2	1
	Adherence to system recommendation	7		2	1

## Discussion

Our systematic review investigated the application of solutions suggested for self-management among transplant patients. Of 40 studies, 32 were devoted to the post-transplantation phase. Other studies have examined the effects of applied solutions in other phases of transplantation. Most studies (72.5%) showed that developed programs and applied solutions had a statistically significant positive impact on the ability of transplanted patients to his/her self-management. In the same way, there is a growing body of evidence regarding the positive results of self-management programs in chronic diseases to enhance a person’s ability to cope with his/her situation and better management of his/her disease [6, 40, 71].

Investigating the most common features and characteristics of applied self-management programs showed that a comprehensive program is needed for effective patient care in SOT. Figure 3 shows different aspects of the main areas of self-management programs for SOT patients. It can be considered as a conceptual model for further research and development of a comprehensive program to enhance patient care.

Because solid organ transplantation is a complex process, patients have little knowledge regarding pre-transplant preparation, transplantation procedure, and post-transplant care and their complications. Therefore, improving patient knowledge of transplantation is a key feature of self-management programs. The literature shows that low health literacy is directly associated with negative outcomes in SOT [72].

Another important aspect of SOT self-management programs is the effective cooperation of the patient with the medical team in reporting their symptoms and following medical advice. One of the most common features of the developed programs is symptom monitoring tools and medication reminders. The applied tools ranged from paper-based diary sheets to web-based electronic forms. However, in the symptom monitoring and medication adherence domains, IT-based interventions were more effective.

The analysis showed that IT-based interventions, including e-health programs for telemonitoring, electronic educational programs, and telerehabilitation programs were the most commonly used solutions in the reviewed studies. Among IT-based solutions, e-health programs or telemedicine-based interventions are more effective than other solutions. Our results are consistent with previous studies that examined the positive effect of IT-based interventions on transplantation in specific organ transplantations [71, 73]. Our investigation showed that non-electronic educational resources should be used alongside other IT-based interventions to promote patient self-care to be more effective and applicable.

In this review, the investigation showed that most IT-based interventions were implemented in the form of m-health applications. Self-management in the form of m-health application with different applicability was effective in terms of clinical outcomes and patient aspects. Previous studies also indicated the effectiveness of m-health applications in enhancing self-management activities.

### Comprehensive self-management program

**Fig. 5:**
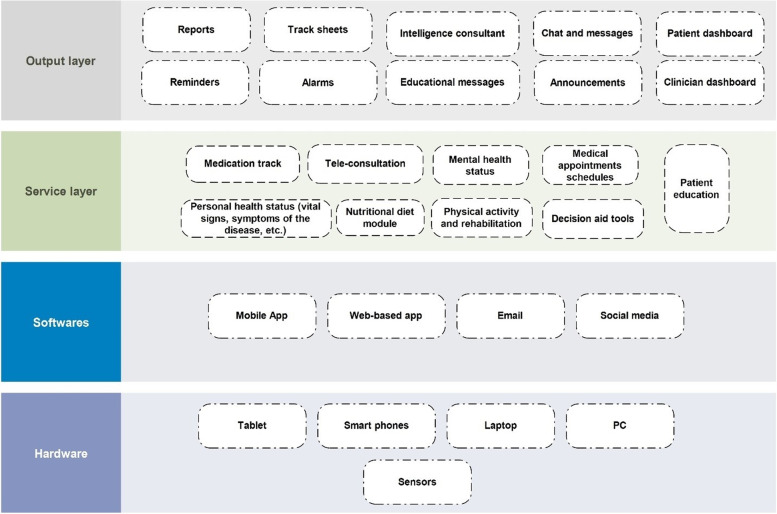
The conceptual model for e-health program to enhance self-management tasks

The results of this review are summarized in a conceptual model [Fig. 5]. We concluded that a pragmatic and effective self-management program should be implemented in the form of an e-health program with various features. It could be one of the best solutions to improve the quality of patient care and move toward patient-centered care.

### Limitations

This study is the first attempt to review and analyze published articles regarding self-management interventions in solid organ transplantation. Some related studies may have been published in the form of letters to the editor, web-based reports, conference papers, or other types of research articles. Thus, we have not considered them based on our exclusion criteria.

The central objective of this study is to examine the devoted solutions with a pragmatic approach. As a result, some concepts that have not been put into practice may not be included in our survey. Also, the interpretation of data depends on the researcher’s perception due to differences and a variety of solutions. Ultimately, the represented framework depends on the researcher's perception of a practical solution and does not offer an ideal response.

The results showed that most studies in this context were conducted by large institutions and reputable organizations. It leads to their data being confounded by the fact that better-funded institutions produce better outcomes. It causes publication bias. Since we want to investigate new and innovative solutions in this context, we limited our research to the last decades. It may lead to some valuable studies being overlooked in the years before 2010. Because children cannot perform self-management daily tasks by themselves, this study was limited to organ transplantation in adults.

## Conclusion

Solid organ transplant patients experience a complex situation in dealing with medical, mental, and social problems from referral time. According to our study, various solutions have been developed for SOT self-management care ranging from paper-based diary sheets to web-based portals to improve patient-centered care.

The results showed that a successful self-management solution to address the patients’ needs must cover various aspects and domains including some features such as continuous symptom recording, reminders, medication log sheets, care assessment tools for healthcare providers, rehabilitation guidance module, and decision support tools. Such programs are used by placing the patient in the center of care while patients are waiting for a new organ or after a transplant. Our findings are valuable for transplantation centers to improve transplantation outcomes by cooperation with their patients to deal with a complex situations with various medical, mental, and social tasks.

## Supplementary Information

**Additional file 1:** **Table A1.** Search strategies in each database.

**Additional file 2.** PRISMA 2020 Main Checklist. 

## Data Availability

The study involves only a review of the literature without involving any data.

## Abbreviations

SOT: Solid Organ Transplantation
PRISMA: Preferred Reporting Items for Systematic Reviews and Meta-Analyses
CASP: Critical Appraisal Program
m-health: Mobile health applications or programs
e-health: Electronic Health
IEEE: Institute of Electrical and Electronics Engineers

## References

[1] Black CK, Termanini KM, Aguirre O, Hawksworth JS, Sosin M (2018). Solid organ transplantation in the 21(st) century. Ann Transl Med.

[2] Vanholder R, Domínguez-Gil B, Busic M, Cortez-Pinto H, Craig JC, Jager KJ, Mahillo B, Stel VS, Valentin MO, Zoccali C (2021). Organ donation and transplantation: a multi-stakeholder call to action. Nat Rev Nephrol.

[3] Lease ED. Primary Care of the Adult Lung Transplant Recipient. In: Wong, C. (eds) Primary Care of the Solid Organ Transplant Recipient. Cham: Springer; 2020. 10.1007/978-3-030-50629-2_7.

[4] Almgren M, Lennerling A, Lundmark M, Forsberg A (2017). Self-efficacy in the context of heart transplantation – a new perspective. J Clin Nurs.

[5] Beckmann S, Künzler-Heule P, Biotti B, Spirig R (2016). Mastering Together the Highs and Lows:Patients’ and Caregivers’ Perceptions of Self-Management in the Course of Liver Transplantation. Prog Transplant.

[6] Jamieson NJ, Hanson CS, Josephson MA, Gordon EJ, Craig JC, Halleck F, Budde K, Tong A (2016). Motivations, Challenges, and Attitudes to Self-management in Kidney Transplant Recipients: A Systematic Review of Qualitative Studies. Am J Kidney Dis.

[7] Vanhoof JMM, Vandenberghe B, Geerts D, Philippaerts P, De Mazière P, DeVito DA, De Geest S, Dobbels F (2018). consortium tP-T: Shedding light on an unknown reality in solid organ transplant patients’ self-management: A contextual inquiry study. Clin Transplant.

[8] O’Connell S, Mc Carthy VJC, Savage E (2018). Frameworks for self-management support for chronic disease: a cross-country comparative document analysis. BMC Health Serv Res.

[9] Aria R, Archer N (2020). An online mobile/desktop application for supporting sustainable chronic disease self-management and lifestyle change. Health Informatics J.

[10] Van de Velde D, De Zutter F, Satink T, Costa U, Janquart S, Senn D, De Vriendt P (2019). Delineating the concept of self-management in chronic conditions: a concept analysis. BMJ Open.

[11] Wiltshire G, Clarke NJ, Phoenix C, Bescoby C (2021). Organ Transplant Recipients' Experiences of Physical Activity: Health, Self-Care, and Transliminality. Qual Health Res.

[12] Yoo HJ, Suh EE (2021). Effects of a smartphone-based self-care health diary for heart transplant recipients: A mixed methods study. Appl Nurs Res.

[13] Bittermann T (2019). Physical Activity After Solid Organ Transplantation: Comprehensive Guidance Is Needed to Advance Future Research Efforts. Transplantation.

[14] Grady PA, Gough LL (2014). Self-management: a comprehensive approach to management of chronic conditions. Am J Public Health.

[15] Udlis KA (2011). Self-management in chronic illness: concept and dimensional analysis. J Nurs Healthc Chronic Illn.

[16] Matarese M, Lommi M, De Marinis MG, Riegel B (2018). A Systematic Review and Integration of Concept Analyses of Self-Care and Related Concepts. J Nurs Scholarsh.

[17] World Health Organization (2014). Regional Office for South-East A: Self care for health.

[18] Richard AA, Shea K (2011). Delineation of Self-Care and Associated Concepts. J Nurs Scholarsh.

[19] Kongsted A, Ris I, Kjaer P, Hartvigsen J (2021). Self-management at the core of back pain care: 10 key points for clinicians. Braz J Phys Ther.

[20] Shamseer L, Moher D, Clarke M, Ghersi D, Liberati A, Petticrew M, Shekelle P, Stewart LA (2015). Preferred reporting items for systematic review and meta-analysis protocols (PRISMA-P) 2015: elaboration and explanation. BMJ.

[21] Institute JB (2014). Joanna Briggs Institute reviewers’ manual.

[22] Moher D, Liberati A, Tetzlaff J, Altman DG (2009). Preferred reporting items for systematic reviews and meta-analyses: the PRISMA statement. BMJ.

[23] Long HA, French DP, Brooks JM (2020). Optimising the value of the critical appraisal skills programme (CASP) tool for quality appraisal in qualitative evidence synthesis. Res Methods Med Health Sci.

[24] Hannes K, Lockwood C, Pearson A (2010). A comparative analysis of three online appraisal instruments' ability to assess validity in qualitative research. Qual Health Res.

[25] Gholamzadeh M, Abtahi H, Ghazisaeeidi M (2021). Applied techniques for putting pre-visit planning in clinical practice to empower patient-centered care in the pandemic era: a systematic review and framework suggestion. BMC Health Serv Res.

[26] Thomas J, Harden A (2008). Methods for the thematic synthesis of qualitative research in systematic reviews. BMC Med Res Methodol.

[27] Xu W, Zammit K (2020). Applying Thematic Analysis to Education: A Hybrid Approach to Interpreting Data in Practitioner Research. Int J Qual Methods.

[28] Schenkel FA, Barr ML, McCloskey CC, Possemato T, O'Conner J, Sadeghi R, Bembi M, Duong M, Patel J, Hackmann AE (2020). Use of a Bluetooth tablet-based technology to improve outcomes in lung transplantation: A pilot study. Am J Transplant.

[29] Van Lint C, Wang W, van Dijk S, Brinkman WP, Rövekamp TJ, Neerincx MA, Rabelink TJ, van der Boog PJ (2017). Self-Monitoring Kidney Function Post Transplantation: Reliability of Patient-Reported Data. J Med Internet Res.

[30] Chen YW, Wei J, Chen HL, Cheng CH, Hou IC (2020). Developing a Heart Transplantation Self-Management Support Mobile Health App in Taiwan: Qualitative Study. JMIR Mhealth Uhealth.

[31] DeVito DA, Song MK, Myers BA, Li R, Hawkins RP, Pilewski JM, Bermudez CA, Aubrecht J, Begey A, Connolly M (2016). A Randomized Controlled Trial of a Mobile Health Intervention to Promote Self-Management After Lung Transplantation. Am J Transplant.

[32] Geramita E, DeVito Dabbs AJ, DiMartini AF, Pilewski JM, Switzer GE, Posluszny DM, Myaskovsky L, Dew MA (2020). Impact of a Mobile Health Intervention on Long-term Nonadherence After Lung Transplantation: Follow-up After a Randomized Controlled Trial. Transplantation.

[33] Gomis-Pastor M, Mirabet Perez S, RoigMinguell E, BrossaLoidi V, Lopez Lopez L, RosAbarca S, Galvez Tugas E, Mas-Malagarriga N, ManguesBafalluy MA (2021). Mobile Health to Improve Adherence and Patient Experience in Heart Transplantation Recipients: The mHeart Trial. Healthcare (Basel).

[34] Jiang Y, Sereika SM, Dabbs AD, Handler SM, Schlenk EA (2016). Acceptance and Use of Mobile Technology for Health Self-Monitoring in Lung Transplant Recipients during the First Year Post-Transplantation. Appl Clin Inform.

[35] Lerret SM, White-Traut R, Medoff-Cooper B, Simpson P, Adib R, Ahamed SI, Schiffman R (2020). Pilot study protocol of a mHealth self-management intervention for family members of pediatric transplant recipients. Res Nurs Health.

[36] McGillicuddy JW, Taber DJ, Mueller M, Patel S, Baliga PK, Chavin KD, Sox L, Favela AP, Brunner-Jackson BM, Treiber FA (2015). Sustainability of improvements in medication adherence through a mobile health intervention. Prog Transplant.

[37] Rosenberger EM, DeVito Dabbs AJ, DiMartini AF, Landsittel DP, Pilewski JM, Dew MA (2017). Long-Term Follow-up of a Randomized Controlled Trial Evaluating a Mobile Health Intervention for Self-Management in Lung Transplant Recipients. Am J Transplant.

[38] Shellmer DA, Dew MA, Mazariegos G, DeVito DA (2016). Development and field testing of Teen Pocket PATH(R), a mobile health application to improve medication adherence in adolescent solid organ recipients. Pediatr Transplant.

[39] Serper M, Barankay I, Chadha S, Shults J, Jones LS, Olthoff KM, Reese PP (2020). A randomized, controlled, behavioral intervention to promote walking after abdominal organ transplantation: results from the LIFT study. Transpl Int.

[40] Ganjali R, Taherzadeh Z, Sabbagh MG, Nazemiyan F, Mamdouhi F, Tabesh H, Aval SB, Golmakani R, Mostafavi SM, Eslami S (2019). Effect of an interactive voice response system on self-management in kidney transplant recipients: Protocol for a randomized controlled trial. Medicine.

[41] Korus M, Cruchley E, Stinson JN, Gold A, Anthony SJ (2015). Usability testing of the Internet program: "teens Taking Charge: Managing My Transplant Online". Pediatr Transplant.

[42] Lieb M, Schiffer M, Erim Y (2020). Optimization of electronically monitored non-adherence in highly adherent renal transplant recipients by reducing the dosing frequency – a prospective single-center observational study. Patient Prefer Adherence.

[43] Lieb M, Weyand M, Seidl M, Erim Y (2020). Prospective single-centre clinical observational study on electronically monitored medication non-adherence, its psychosocial risk factors, and lifestyle behaviours after heart transplantation: a study protocol. Bmj Open.

[44] Nielsen C, Agerskov H, Bistrup C, Clemensen J (2020). User involvement in the development of a telehealth solution to improve the kidney transplantation process: A participatory design study. Health Informatics J.

[45] Tian M, Wang B, Xue Z, Dong D, Liu X, Wu R, Yu L, Xiang J, Zhang X, Zhang X (2021). Telemedicine for Follow-up Management of Patients After Liver Transplantation: Cohort Study. JMIR Med Inform.

[46] Wang W, van Lint CL, Brinkman WP, Rövekamp TJM, van Dijk S, van der Boog PJM, Neerincx MA (2017). Renal transplant patient acceptance of a self-management support system. BMC Med Inform Decis Mak.

[47] Dahl KG, Wahl AK, Urstad KH, Falk RS, Andersen MH (2021). Changes in Health Literacy during the first year following a kidney transplantation: Using the Health Literacy Questionnaire. Patient Educ Couns.

[48] Kayler LK, Dolph B, Seibert R, Keller M, Cadzow R, Feeley TH (2020). Development of the living donation and kidney transplantation information made easy (KidneyTIME) educational animations. Clin Transplant.

[49] Kayler LK, Majumder M, Bonner K, Ranahan M, Dolph B, Cadzow R, Feeley TH (2020). Development and preliminary evaluation of an animation (simplifyKDPI) to improve kidney transplant candidate understanding of the Kidney Donor Profile Index. Clin Transplant.

[50] Kim S, Ju MK, Son S, Jun S, Lee SY, Han CS (2020). Development of video-based educational materials for kidney-transplant patients. PLoS ONE.

[51] Mansell H, Rosaasen N, West-Thielke P, Wichart J, Daley C, Mainra R, Shoker A, Liu J, Blackburn D (2019). Randomised controlled trial of a video intervention and behaviour contract to improve medication adherence after renal transplantation: the VECTOR study protocol. BMJ Open.

[52] Hickman IJ, Coran D, Wallen MP, Kelly J, Barnett A, Gallegos D, Jarrett M, McCoy SM, Campbell KL, Macdonald GA (2019). 'Back to Life'-Using knowledge exchange processes to enhance lifestyle interventions for liver transplant recipients: A qualitative study. Nutr Diet.

[53] Leek RB, Park JM, Koerschner C, Mawby J, Sonnenday CJ, Wright Nunes JA, Sharma P (2019). Novel educational and goal-setting tool to improve knowledge of chronic kidney disease among liver transplant recipients: A pilot study. PLoS ONE.

[54] Urstad KH, Øyen O, Andersen MH, Moum T, Wahl AK (2012). The effect of an educational intervention for renal recipients: a randomized controlled trial. Clin Transplant.

[55] Frank-Bader M, Beltran K, Dojlidko D (2011). Improving transplant discharge education using a structured teaching approach. Progress in transplantation (Aliso Viejo, Calif).

[56] Evald L, Graarup J, Højskov IE (2020). Diary for self-observation: A self-management tool for recipients of lung transplantation-A pilot study. Nurs Open.

[57] Bailey DE, Hendrix CC, Steinhauser KE, Stechuchak KM, Porter LS, Hudson J, Olsen MK, Muir A, Lowman S, DiMartini A (2017). Randomized trial of an uncertainty self-management telephone intervention for patients awaiting liver transplant. Patient Educ Couns.

[58] Been-Dahmen JMJ, Beck DK, Peeters MAC, Van Der Stege H, Tielen M, Van Buren MC, Ista E, Van Staa A, Massey EK (2019). Evaluating the feasibility of a nurse-led self-management support intervention for kidney transplant recipients: A pilot study. BMC Nephrology.

[59] Li L, Ma Z, Wang W (2020). Influence of transitional care on the self-care ability of kidney transplant recipients after discharge. Ann Palliat Med.

[60] Hsiao CY, Lin LW, Su YW, Yeh SH, Lee LN, Tsai FM (2016). The Effects of an Empowerment Intervention on Renal Transplant Recipients: A Randomized Controlled Trial. J Nurs Res.

[61] Jerson B, D'Urso C, Arnon R, Miloh T, Iyer K, Kerkar N, Annunziato RA (2013). Adolescent transplant recipients as peer mentors: a program to improve self-management and health-related quality of life. Pediatr Transplant.

[62] Schaevers V, Schoonis A, Frickx G, Verleden G, Jans C, Rosseel C, Meelberghs M, Reinquin I, Dobbels F, Interdisciplinary Lung T (2012). Implementing a standardized, evidence-based education program using the patient's electronic file for lung transplant recipients. Prog Transplant.

[63] Freier C, Oldhafer M, Offner G, Dorfman S, Kugler C (2010). Impact of computer-based patient education on illness-specific knowledge and renal function in adolescents after renal transplantation. Pediatr Transplant.

[64] Harrison JJ, Badr S, Hamandi B, Kim SJ (2017). Randomized controlled trial of a computer-based education program in the home for solid organ transplant recipients: Impact on medication knowledge, satisfaction, & adherence. Transplantation.

[65] Han A, Min S-I, Ahn S, Min S-K, Hong H-J, Han N, Kim YS, Ahn C, Ha J (2019). Mobile medication manager application to improve adherence with immunosuppressive therapy in renal transplant recipients: A randomized controlled trial. PLOS ONE.

[66] Côté J, Fortin MC, Auger P, Rouleau G, Dubois S, Vaillant I, Gélinas-Lemay É, Boudreau N (2019). Web-Based Tailored Nursing Intervention to Support Medication Self-management: A Qualitative Study of the Experience of Kidney Transplant Recipients. Comput Inform Nurs.

[67] Wickerson L, Helm D, Gottesman C, Rozenberg D, Singer LG, Keshavjee S, Sidhu A (2021). Telerehabilitation for Lung Transplant Candidates and Recipients During the COVID-19 Pandemic: Program Evaluation. JMIR Mhealth Uhealth.

[68] Williams FR, Vallance A, Faulkner T, Towey J, Durman S, Kyte D, Elsharkawy AM, Perera T, Holt A, Ferguson J (2019). Home-Based Exercise in Patients Awaiting Liver Transplantation: A Feasibility Study. Liver Transpl.

[69] Kim J, Kim K, Jang I (2019). Symptom Experience, Self-Care Adherence, and Quality of Life Among Heart Transplant Recipients in South Korea. Clin Nurs Res.

[70] Weng LC, Dai YT, Huang HL, Chiang YJ (2010). Self-efficacy, self-care behaviours and quality of life of kidney transplant recipients. J Adv Nurs.

[71] Eslami S, Khoshrounejad F, Golmakani R, Taherzadeh Z, Tohidinezhad F, Mostafavi SM, Ganjali R (2021). Effectiveness of IT-based interventions on self-management in adult kidney transplant recipients: a systematic review. BMC Med Inform Decis Mak.

[72] Chisholm-Burns MA, Spivey CA, Pickett LR (2018). Health literacy in solid-organ transplantation: a model to improve understanding. Patient Prefer Adherence.

[73] Shahmoradi L, Abtahi H, Amini S, Gholamzadeh M (2020). Systematic review of using medical informatics in lung transplantation studies. Int J Med Inform.

