# Supplementary material for: Pragmatic solutions to enhance self-management skills in solid organ transplant patients: systematic review and thematic analysis
Source: BMC Prim Care. 2022 Jun 30;23:166. doi: 10.1186/s12875-022-01766-z (PMC9247970; doi:10.1186/s12875-022-01766-z)
Supplement: Supplementary file 1 — Additional file 1: Table A1. Search strategies in each database. [file 12875_2022_1766_MOESM1_ESM.docx]

**Additional file 1**

Table A-1- Search strategies in each database

| **Database** | **Search strategies** | **Results (Count of studies)** |
| --- | --- | --- |
| PubMed | (("Organ Transplantation"[Title/Abstract]) OR ("Organ Grafting"[Title/Abstract]) OR ("Organ Transplantations"[Title/Abstract]) OR ("Organ Transplantation"[Mesh])) AND (("Self Administration"[Mesh]) OR ("Self-Management"[Mesh]) OR ("Self Administration"[Title/Abstract]) OR ("Self-Management"[Title/Abstract]) OR ("Self Management"[Title/Abstract]) OR ("self efficacy"[Title/Abstract]) OR ("Self Efficacy"[Mesh]) OR ("Self care"[Title/Abstract])) AND (2010:2021[pdat]) | 277 |
| Scopus | ( TITLE-ABS-KEY ( "Self Efficacy" ) )  OR  ( TITLE-ABS-KEY ( "Self Management" ) )  OR  ( TITLE-ABS-KEY ( "Self-Management" ) )  OR  ( TITLE-ABS-KEY ( "Self Administration" ) )  OR  ( TITLE-ABS-KEY ( "Self-Management" ) )  OR  ( TITLE-ABS-KEY ( "Self-Efficacy" ) )  OR  ( TITLE-ABS-KEY ( "self-care" ) )  OR  ( TITLE-ABS-KEY ( "self care" ) )  AND ( ( TITLE-ABS-KEY ( ( *"Organ Transplantation"* ) )  OR  TITLE-ABS-KEY ( ( *"Organ Grafting"* ) )  OR  TITLE-ABS-KEY ( ( *"Organ Transplantations"* ) ) )  AND  PUBYEAR  >  *2009* )  AND  ( ( TITLE-ABS-KEY ( *"Self Efficacy"* ) )  OR  ( TITLE-ABS-KEY ( *"Self Management"* ) )  OR  ( TITLE-ABS-KEY ( *"Self-Management"* ) )  OR  ( TITLE-ABS-KEY ( *"Self Administration"* ) )  OR  ( TITLE-ABS-KEY ( *"Self-Management"* ) )  OR  ( TITLE-ABS-KEY ( *"Self-Efficacy"* ) )  OR  ( TITLE-ABS-KEY ( *"self-care"* ) )  OR  ( TITLE-ABS-KEY ( *"self care"* ) ) )  AND  ( LIMIT-TO ( DOCTYPE,  *"ar"* ) )  AND  ( LIMIT-TO ( LANGUAGE,  *"English"* ) ) | 55 |
| Web of Sciences | 1.TS = (("Organ Transplantation") OR ("Organ Grafting") OR ("Organ Transplantations") OR ("Organ Transplantation"))  From 2010 to 2020  TS= (("Self Efficacy") OR ("Self Management") OR ("Self-Management") OR ("Self Administration") OR ("Self-Management") OR ("Self-Efficacy") OR ("self-care") OR ("self care"))  1 AND 2 | 48 |
| PsycInfo OVID | (("transplantation".mp. [mp=title, abstract, full text, caption text]) Or ("organ transplantation".mp. [mp=title, abstract, full text, caption text]) or ("transplantation".af.)) AND (("("Self-Efficacy".mp. [mp=title, abstract, full text, caption text]) or ("Self Management".mp. [mp=title, abstract, full text, caption text]) or ("Self-Management ".mp. [mp=title, abstract, full text, caption text]) or ("Self Administration".mp. [mp=title, abstract, full text, caption text]) or ("Self care".mp. [mp=title, abstract, full text, caption text]) or or ("Self-care".mp. [mp=title, abstract, full text, caption text])) to (yr="2010 -Current" and original articles) | 131 |
| ScienceDirect | (("Organ Transplantation") OR ("Organ Grafting") OR ("Organ Transplantations") OR ("Organ Transplantation")) AND (("Self Administration") OR ("Self-Management") OR ("Self Administration") OR ("Self-Management") OR ("Self Management") OR ("self efficacy") OR ("Self Efficacy") OR ("Self care")) | 154 |
